# Antioxidant icariside II combined with insulin restores erectile function in streptozotocin-induced type 1 diabetic rats

**DOI:** 10.1111/jcmm.12480

**Published:** 2015-03-17

**Authors:** Lin Wang, Yongde Xu, Huixi Li, Hongen Lei, Ruili Guan, Zhezhu Gao, Zhongcheng Xin

**Affiliations:** Andrology Center, Peking University First Hospital, Peking UniversityBeijing, China

**Keywords:** diabetes mellitus, erectile dysfunction, icariside II, metabolic memory

## Abstract

Erectile dysfunction (ED) worsens in patients with diabetes mellitus (DM) despite good control of blood glucose level with insulin. Recent studies imply that diabetic vascular stresses (*e.g*. oxidative stress) persist in spite of glucose normalization, which is defined as metabolic memory. Studies suggest that the interaction between advanced glycation end products (AGEs) and their receptor (RAGE) mediates the development of metabolic memory. To investigate the effects of the antioxidant icariside II plus insulin on erectile function in streptozotocin (STZ)- induced type 1 diabetic rats. Fifty 8-week-old Sprague-Dawley rats were randomly distributed into five groups: normal control, diabetic, insulin-treated diabetic, icariside II-treated diabetic, and insulin plus icariside II-treated diabetic. Diabetes was induced by a single intraperitoneal injection of STZ. Eight weeks after induction of diabetes, icariside II was administered by gastric lavage once a day (5 mg/kg) for 6 weeks; and 2–6 units of intermediate-acting insulin were given to maintain normal glycemia for 6 weeks. The main outcome measures were the ratio of intracavernous pressure (ICP) to mean arterial pressure (MAP); histology of penile endothelial cells and smooth muscle cells; neural nitric oxide synthase, AGEs and RAGE expression; malondialdehyde concentration; superoxide dismutase activity; and apoptosis index. Diabetic rats demonstrated a significantly lower ICP/MAP ratio, reduced penile endothelial cells, reduced smooth muscle cells, increased AGEs and RAGE, and increased apoptosis. Insulin and icariside II monotherapy partially restored erectile function and histological changes. However, the combination therapy group showed significantly better erectile parameters, cytological components and biochemistry, similar to those in the normal control group. These results suggest that, although insulin can effectively control glycemic levels, it does not completely alter the pathological changes in erectile tissues. Better efficacy could be expected with tight glycemic control plus the antioxidant icariside II. The proposed combination therapy might have the potential to eliminate metabolic memory by down-regulating the AGEs-RAGE-oxidative stress axis.

## Introduction

Erectile dysfunction (ED) is a prevalent disease with a significant impact on quality of life. Depending on the definition used and study design prevalence varies from 10 to 52%, mainly in men between 40 and 70 years, with an incidence in western countries between 25 and 30 new cases per 1000 inhabitants year 1. Furthermore, multiple studies have demonstrated the roles organic factors play in the development of ED 2. Diabetes mellitus (DM) is a significant risk factor in the development of ED. Consequently, DM-associated ED is the most common type of ED 3, and the risk of developing ED is 1.9–4 times higher in diabetic *versus* non-diabetic men 4. Furthermore, ED is considered an early manifestation of atherosclerosis and a precursor of systemic vascular disease 5. Although blood glucose levels can be controlled by insulin, patients with DM continue to suffer from numerous sequelae of their disease, including ED.

Patients with type 1 DM develop endothelial dysfunction even after normal glycemia is achieved 6. Furthermore, several studies suggest that hyperglycaemia induces endothelial dysfunction through the generation of oxidative stress 7. Kowluru *et al*. examined the retinas of diabetic rats with 6 months of poor glycemic control followed by 6 months of good glycemic control with insulin 8 and demonstrated increased levels of oxidative and nitrative stress in rats with poor glycemic control. The increased oxidative stress was associated with a persistent reduction in the activity of the antioxidant enzyme superoxide dismutase (SOD). These rats also demonstrated an increase in acellular capillaries, suggesting ongoing retinal microvascular injury. This phenomenon, whereby diabetic vascular stresses persist in spite of glucose normalization, is defined as metabolic memory. Reactive derivatives of non-enzymatic glucose-protein condensation reactions comprise a heterogeneous group of irreversible adducts called advanced glycation end-products (AGEs). There is a growing body of evidence to suggest that the interaction between AGEs and their receptor (RAGE) elicits oxidative stress and mediates the development of metabolic memory 9.

Plants of the genus *Epimedium* (herba epimedii) have been utilized for the treatment of ED in traditional Chinese medicine for centuries. Icariside II (ICA II; C_27_H_32_O_10_, 514.54 kD) is a type of flavonol isolated from herba epimedii. Zhou *et al*. showed that ICA II had therapeutic effects in diabetes-related ED, which was modulated by preservation/recovery of smooth muscle, endothelial function and NOS activity 10.

The aims of this study were to investigate the correlation between AGEs/RAGE-mediated oxidative stress and erectile function and to examine the effect of antioxidant ICA II plus insulin on improvement of erectile function in streptozotocin (STZ)-induced type 1 diabetic rats.

## Materials and methods

### Animals

Fifty 8-week-old Sprague–Dawley rats were randomly distributed into five groups: normal control rats (C), diabetic control rats (D), insulin-treated diabetic rats (I), ICA II-treated diabetic rats (D+ ICA II) and insulin plus ICA II-treated diabetic rats (D+I+ ICA II). All experiments were approved by the Institutional Animal Care and Use Subcommittee of Peking University First Hospital. To establish diabetes, rats were fasted for 16 hrs and underwent intraperitoneal (IP) injection of freshly prepared STZ at 60 mg/kg (Sigma Chemical Co, St. Louis, MO, USA) or sham vehicle [0.1 mol/l citrate-phosphate buffer (pH 4.5)], as performed by Usta *et al*. 11. 72 hrs following STZ or vehicle injection, tail blood samples were obtained from fasting rats and blood glucose levels were measured using a blood glucometer (B. Braun, Melsungen, Germany). Only rats with fasting blood glucose concentrations ≥300 mg/dl were included in the diabetic groups. Per previous studies, significant reduction in erectile function is not observed until 8 weeks after the induction of diabetes 12. Thus, 8 weeks after diabetes induction, ICA II at 5 mg/kg was administered by gastric lavage once a day. We also injected 2–6 units of intermediate-acting insulin (Novo Nordisk, Bagsvaerd, Denmark) subcutaneously along the dorsal aspect twice a day to achieve normal blood glucose levels 13. After 6 weeks of treatment, erectile function was evaluated by cavernous nerve electrostimulation, and the penis was harvested for histological examination and western blot analysis.

### Measurement of Glycated Haemoglobin (HbA1c)

Glycated haemoglobin was measured using six whole-blood samples from each group. Haemoglobin A1c (HbA1c) was measured by high-performance liquid chromatography (HPLC; Ultra2, Primus Corp., Kansas City, MO, USA) in the diabetes laboratory of our institution.

### Measurement of Erectile Function

The rats from each group were anesthetized with 5% sodium pentobarbital injected IP. The major pelvic ganglion, cavernous nerves, and pelvic organs were exposed through a lower midline laparotomy incision. The skin overlying the penis was removed and the penile crus was exposed by removing part of the overlying ischiocavernous muscle. Two 24-gauge needles connected to PE-50 tubes with heparinized saline (250 IU/ml) were carefully inserted into the crus and left carotid artery. The other end of the PE-50 tube was connected to a data acquisition system (MP150; BIOPAC Systems Inc, Goleta, CA, USA). The cavernous nerve was exposed and electrostimulation (12 Hz, pulse width 5 msec., 5 V, duration 60 sec.) was applied with a stainless bipolar needle electrode. Mean arterial pressure (MAP) was measured simultaneously, calculated according to the formula: diastolic blood pressure + [(systolic blood pressure − diastolic blood pressure)/3]. The ratio of maximal intracavernous pressure (ICP [mmHg]) to MAP (mmHg)] was calculated to normalize for variations in systemic blood pressure.

### Immunohistochemistry

Immunohistochemistry was performed on 5-μm sections that were deparaffinized and hydrated by sequential incubations in xylene and ethanol. Specimens were washed three times in PBS for 5 min. and blocked with 3% hydrogen peroxide for 10 min. After blocking with 3% goat serum in PBS for 30 min., the slides were incubated with primary antibody overnight (AGEs 1:3000; Abcam, Cambridge, MA, USA). Slides were incubated with secondary antibody followed by staining with the Histo-stain-Plus kit (Zymed Laboratories, South San Francisco, CA, USA). Negative controls were performed by omission of the primary antibody. The mean density of AGEs staining was evaluated by Image J (National Institutes of Health, Bethesta, MD, USA).

### Western blot

One half of the rat penis was processed for total protein extraction by mechanical homogenization in cold lysis buffer, phosphate buffer solution, and 0.1% Triton X-100 in the presence of protease inhibitors (protease inhibitor cocktail; P8340, Sigma-Aldrich, St. Louis, MO, USA). After centrifugation (89440g, 5 min.), supernatants were collected and proteins were quantified using the BCA Protein Assay Kit (Thermo Scientific, Waltham, MA, USA). 20 μg of each sample was separated by electrophoresis in a 10% sodium dodecyl sulphate-polyacrylamide gel. After transfer, the membranes were incubated with rabbit anti-rat RAGE antibodies (1:2000 dilution; Abcam), rabbit anti-rat alpha smooth muscle actin antibodies (1:2000 dilution; Abcam), rabbit anti-rat eNOS antibodies (1:400 dilution; Abcam) and rabbit anti-rat neural nitric oxide synthase (nNOS) antibody (1:400 dilution; Abcam). Antibody binding was detected after incubation with secondary horseradish-peroxidase-coupled antibody (1:2000; Santa Cruz Biotechnology, Santa Cruz, CA, USA) with an enhanced chemiluminescence kit (Amersham Biosciences, Little Chalfont, UK). β-actin and GAPDH were used as internal control. Membrane was stripped and re-probed with goat anti-rat β-actin antibodies (1:2000; Santa Cruz Biotechnology) and goat anti-rat GAPDH antibodies (1:1000; Santa Cruz Biotechnology) as described above. The resulting images were analyzed with Image J (National Institutes of Health) to determine the integrated density of each protein band.

### SOD activities and malondialdehyde concentrations in rat penis

Part of the supernatants collected for western blot was used for measuring SOD activity and malondialdehyde (MDA) concentration, a breakdown product of reactive oxygen species 14. SOD activity was measured using an assay kit with nitroblue tetrazolium substrate according to the manufacturer's instructions (Beyotime, Shanghai, China). Absorbance was read at 550 nm and the activity was calculated using the formula: [(control value - blank value) - (sample value - blank value)]/(control value - blank value)/(control value). SOD activity was given as U/mg protein. MDA concentration was measured with the thiobarbituric acid (TBA) reaction method. Briefly, the supernatant fraction was mixed with TBA reagent consisting of 0.375% TBA and 15% trichloroacetic acid in 0.25 mM hydrochloric acid. The reaction mixtures were placed in boiling water, and then the absorbance of the supernatant was measured at 535 nm. MDA levels were expressed as nmol/mg protein 15.

### Immunofluorescence

At the conclusion of the erectile function evaluation, rats were killed and their penises were harvested for histology. The penis was cut into two halves: one half was stored in liquid nitrogen for protein extraction, the other half was fixed with 2% formaldehyde and 0.002% picric acid in 0.1 M PBS for 4 hrs, followed by immersion in 30% sucrose in PBS overnight at 4°C. The fixed tissue was then embedded in optimal cutting temperature compound (Sakura Finetek, Torrance, CA, USA), cut into 5-μm thick sections, mounted on glass slides (four sections per slide), and subjected to immunofluorescent (IF) staining. For IF staining, the slides were placed in 0.3% H_2_O_2_/methanol for 10 min., washed twice in PBS for 5 min. and incubated with 3% goat serum in PBS/0.3% Triton X-100 for 30 min. at room temperature. After draining this solution from the tissue section, the slides were incubated at 4°C with rabbit anti-nNOS (1:100, SC-648; Santa Cruz Biotechnology) or rabbit anti-vWF (vWF; 1:1000; Abcam) antibody overnight. Control tissue sections were similarly prepared except no primary antibody was added. After rinses with PBS, the sections were incubated with AlexaFluor-594-conjugated secondary antibody (Invitrogen, Carlsbad, CA, USA). Smooth muscle was stained by incubation with FITC-conjugated phalloidin (Sigma-Aldrich) for 30 min. at room temperature. Nuclei were stained with 4′,6-diamidino-2-phenylindole (DAPI; Invitrogen).

### Apoptosis assay

Tissue sections were fixed with 4% paraformaldehyde/PBS then subjected to TUNEL assay with a one-step TUNEL apoptosis assay kit (Beyotime). The percentage of TUNEL-positive cells was determined in a blinded manner. Briefly, the cells were permeabilized with 0.1% Triton X-100 for 3 min. on ice, and then subjected to the TUNEL assay for 1 hr at 37°C in the dark. Negative controls were incubated in the absence of the terminal transferase. Finally, cells were counterstained with DAPI. Slides were observed with a DMI 6000B fluorescence microscope (Leica, Wetzlar, Germany) with 550-nm excitation and 570-nm emission. The cells with red fluorescence were defined as apoptotic cells.

### Statistical analysis

The results were expressed as mean ± SD. One-way anova followed by Student Newmann–Keuls *t*-test was used for comparisons using Prism 5 software (GraphPad, La Jolla, CA, USA). Values of *P* < 0.05 were considered statistically different.

## Results

### Metabolic variables and erectile function assessment

**Figure 1:**
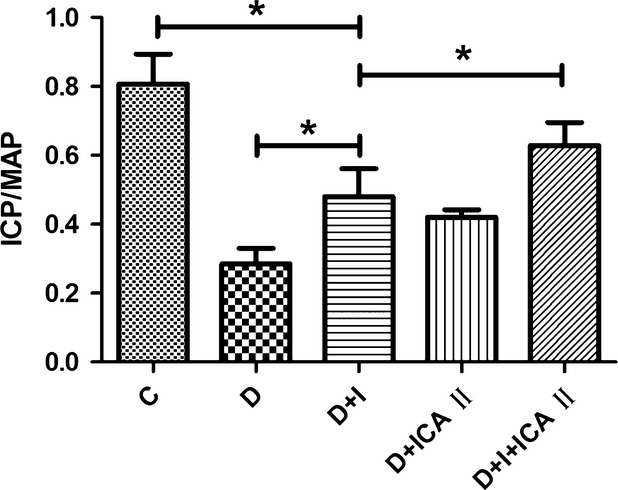
Evaluation of erectile function. Erectile function was measured in control (C), diabetic (D), D+ insulin (I), D+ICA II, and D+I+ICA II groups (*n* = 10 in each group). Erectile function was measured *via* increased intracavernous pressure (ICP) in response to electrostimulation of the major pelvic ganglion. ICP was normalized to mean arterial pressure (MAP). MAP is 160 cmH_2_O. * denotes *P* < 0.05 when comparing the two groups under each end of the capped line.

Fourteen weeks after diabetes was induced, random glucose concentrations and HbA1c (%) of the diabetic group were significantly higher than those of the age-matched normal group. Bodyweight was significantly lower in the diabetic rats compared to the normal group. No statistical difference in bodyweight, glucose concentration or HbA1c (%) was found between the normal group and the insulin-treated diabetic group. Also, there was no statistical difference in bodyweight, glucose concentration or HbA1c (%) between the normal group and the insulin plus icariside II-treated group (Table1). The ratio of ICP to MAP was significantly lower in the diabetic rats, and was only partially improved by insulin or icariside II monotherapy. The insulin plus icariside II treatment group showed better erectile function compared to the groups receiving insulin or icariside II only (Fig.1). Insulin plus icariside II produced near normal erectile parameters (Fig.1). No statistical difference in MAP was noted among the groups.

**Table 1:** Metabolic and physiological variables

Variable	C	D	D+I	D+ICAII	D+I+ICAII
Initial weight (g)	292.5 ± 11.0	293.0 ± 14.4	277.1 ± 13.9	280.5 ± 16.5	292.5 ± 14.4
Final weight (g)	553 ± 47.6	359.8 ± 28.5*	498.3 ± 37.8†	363.1 ± 24.9*	499.1 ± 29.7†
Initial glucose (mmol/l)	7.4 ± 0.9	6.9 ± 1.1	7.1 ± 1.3	7.4 ± 0.8	6.9 ± 0.8
Final glucose (mmol/l)	7.2 ± 1.1	24.9 ± 6.2*	6.8 ± 2.1†	22.9 ± 4.0*	7.0 ± 1.4†
HbA1C (%)	4.4 ± 0.2	10.1 ± 0.7*	4.6 ± 0.5†	9.5 ± 1.3*	4.8 ± 0.5†

Values are expressed as mean ± sd for *n* = 10 animals per group.

C, normal control; D, diabetic control; I, intermediate-acting insulin; ICA II, Icariside II.

* *P* < 0.01 when compared with the normal control group.

† *P* < 0.01 when compared with the diabetic group.

### Icariside II or insulin monotherapy partially restores endothelial, nerve and smooth muscle components, whereas combination therapy restores these components to near normal levels

**Figure 2:**
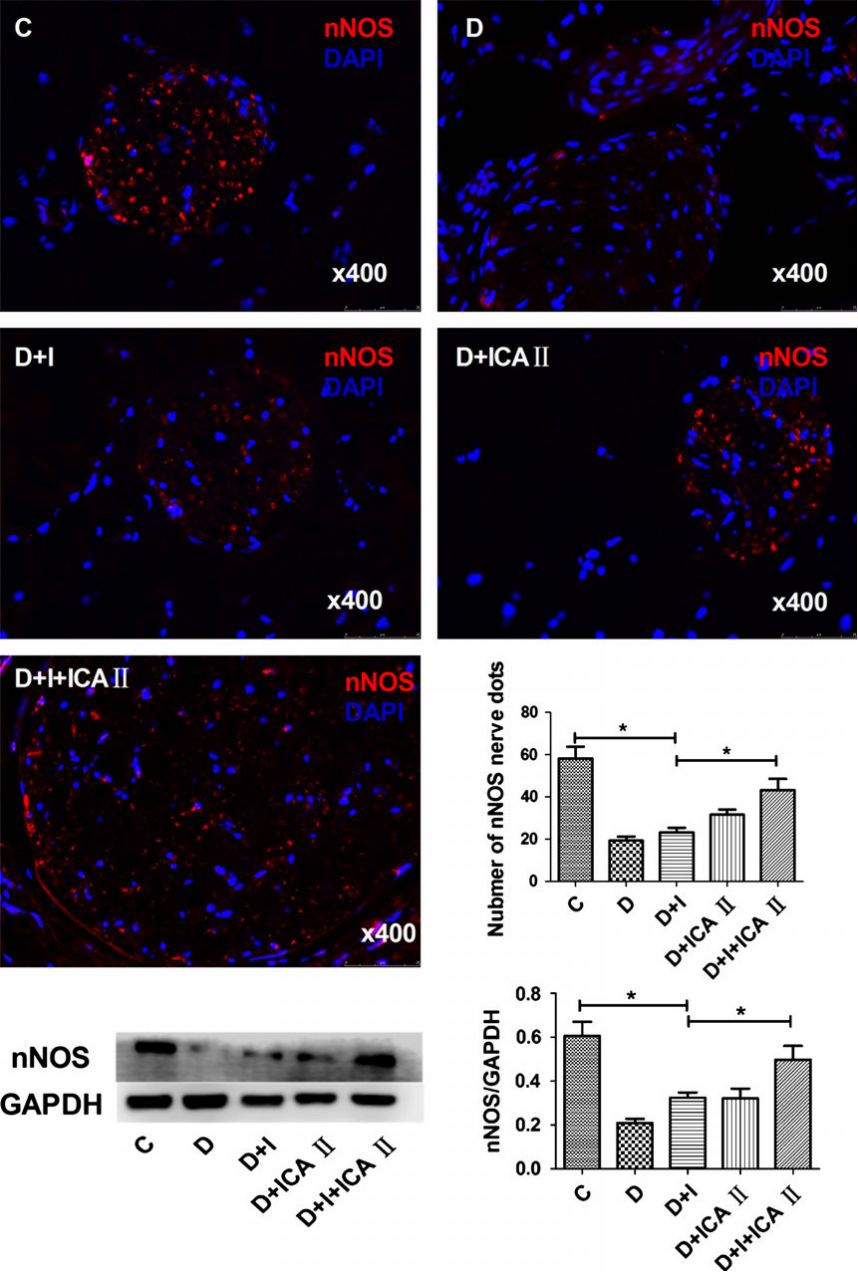
Evaluation of nNOS expression. Penile tissues were examined for nNOS expression in nerve endings. The results are shown in the representative histological images with the red and blue (DAPI) stains indicating nNOS-positive nerves and cell nuclei, respectively. Quantitative data of nNOS expression in each group are shown in the bar graph. Rats in the combination D+I+ICA II group demonstrated significantly increased nNOS compared to diabetic (D) rats, which neared the level of the control (C) group. The insulin (I) and ICA II monotherapy groups demonstrated partially increased nNOS expression. Western blot result of nNOS showed similar trend to immunofluorescence. * denotes *P* < 0.05 when comparing the two groups under each end of the capped line.

**Figure 3:**
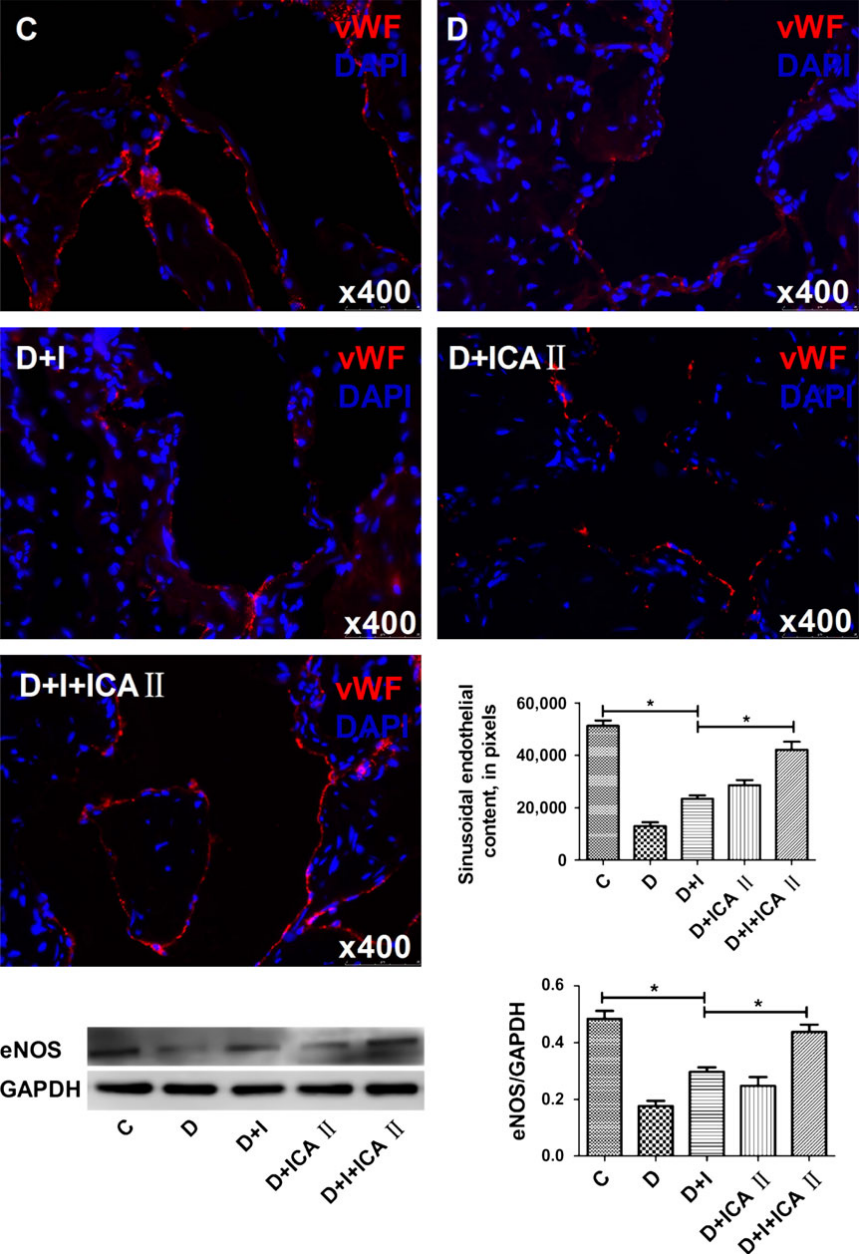
Evaluation of endothelial content. Penile tissues were examined for von Willebrand Factor (vWF) expression, a marker of endothelial cells. The results are shown in the representative histological images with red (vWF) and blue (DAPI) stains indicating the endothelium and cell nuclei, respectively. Quantitative data of vWF expression in cavernous sinusoids are shown in the bar graph. Rats in the combination D+I+ICA II group demonstrated significantly increased vWF expression compared to diabetic (D) rats, nearing the level of the control (C) group. The insulin (I) and ICA II monotherapy groups demonstrated partially increased vWF expression. Western blot result of eNOS showed similar trend to immunofluorescence. * denotes *P* < 0.05 when comparing the two groups under each end of the capped line.

**Figure 4:**
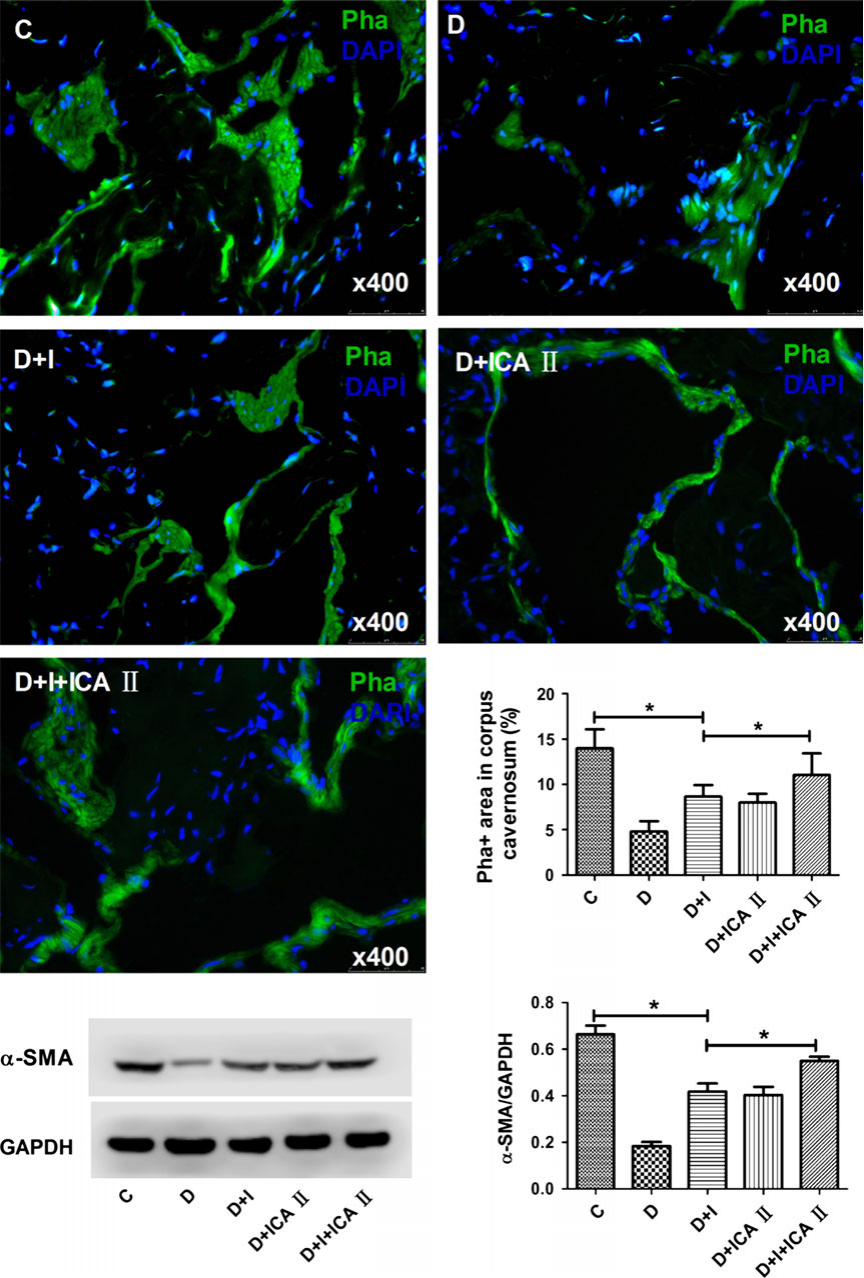
Evaluation of smooth muscle content. Penile tissues were examined for phalloidin (Pha) staining, a marker of smooth muscle expression, and DAPI, a marker for cell nuclei. The results are shown in the representative histological images with the green and blue stains indicating smooth muscle and cell nuclei, respectively. Quantitative data for cavernous smooth muscle content are shown in the bar graph. Rats in the combination D+I+ICA II group demonstrated significantly increased Pha compared to diabetic (D) rats, nearing that of the control (C) group. The insulin (I) and ICA II monotherapy groups demonstrated partially increased Pha expression. Western blot result of α-SMA showed similar trend to immunofluorescence.* denotes *P* < 0.05 when comparing the two groups under each end of the capped line.

Rats in the diabetic group showed a significant decrease in nNOS in dorsal nerves, which was partially restored by insulin (Fig.2). Likewise, rats in the diabetic group showed significant reductions in endothelial (Fig.3) and cavernous smooth muscle (Fig.4) contents, which were partially restored by insulin. However, insulin plus icariside II combination therapy restored these components to near normal levels (*P* > 0.05; Figs4).

### Icariside II reduces RAGE expression but has no effect on AGEs level, whereas combination therapy restores RAGE expression to a near normal level

**Figure 5:**
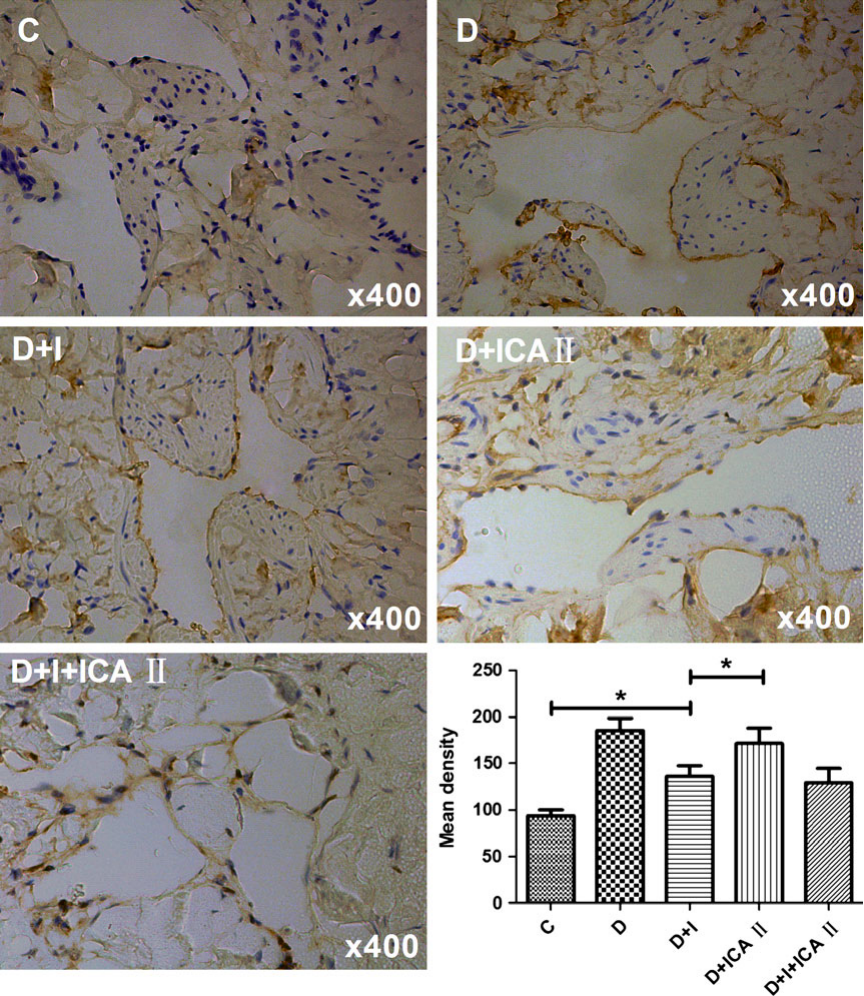
Evaluation of AGEs deposition. Representative immunohistochemistry images for AGEs staining in the corpus cavernosum from each group. AGEs deposition is demonstrated in penile corpus cavernosum, and predominantly in the endothelium and smooth muscle. * denotes *P* < 0.05 when comparing the two groups under each end of the capped line. C, control group; D, diabetic group; I, insulin group; ICA II, icariside II.

**Figure 6:**
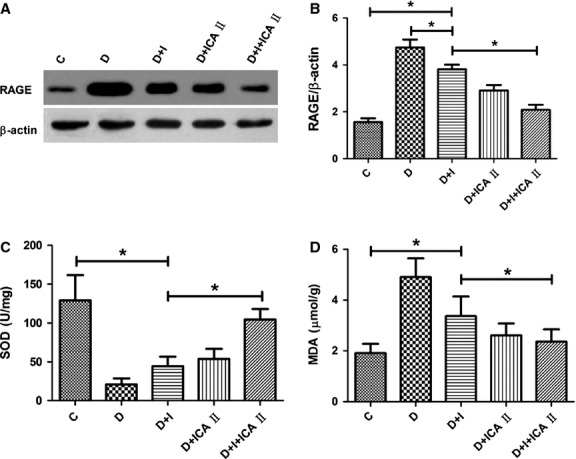
(A and B) Evaluation of RAGE expression by Western blot. Representative Western blots showing RAGE and β-actin protein levels in corpus cavernosum tissue of five groups. Quantification of protein levels using RAGE/β-actin was expressed as mean ± SD. RAGE expression is normalized in the ICA II monotherapy and combination therapy groups. * denotes *P* < 0.05 when comparing the two groups under each end of the capped line. C, control group; D, diabetic group; I, insulin group; ICA II, icariside II. (C) Evaluation of SOD activity. SOD activity in corpus cavernosum tissue from each group. Combination therapy normalized SOD activity, bringing it close to that of the control rats. * denotes *P* < 0.05 when comparing the two groups under each end of the capped line. (D) Evaluation of malondialdehyde (MDA) content. MDA levels in corpus cavernosum tissue from each group are shown. Combination therapy decreased the MDA level to that of control rats. * denotes *P* < 0.05 when comparing the two groups under each end of the capped line.

Rats in the diabetic group showed large amounts of AGEs deposition in the corpus cavernosum, especially in endothelial cells of sinusoids. Although insulin can control blood glucose to normal levels, there was still considerable AGEs deposition in the diabetic group. Icariside II had no effect on the deposition of AGEs in tissues (Fig.5). Diabetic rats had a significant increase in RAGE expression, with a similar distribution within the penis to that observed for AGEs immunostaining, and this increase was not completely attenuated by insulin. Icariside II significantly reduced RAGE expression compared with untreated diabetic animals. Likewise, combination therapy reversed RAGE expression to a level similar to that of the normal control group (Fig.6A and B).

### Icariside II and insulin each partially restores SOD activity and lowers lipid peroxidation levels, whereas combination therapy restores the two parameters to control levels

Rats in the diabetic group showed a significant decline in SOD activity and an increase in MDA concentration, which is an indicator of lipid peroxidation level. Rats treated with insulin alone showed control of blood glucose to normal levels, but still had decreased SOD activities and increased MDA compared to normal controls. Rats treated with insulin plus icariside II had higher SOD activity and lower lipid peroxidation levels compared to the insulin-treated group (*P* < 0.05). Moreover, the combination therapy group showed no statistical differences in SOD activity and MDA level when compared to the control group (*P* > 0.05; Fig.6C and D).

### Icariside II and insulin each partially reduces apoptosis, but the combination therapy reduces apoptosis to a larger extent

**Figure 7:**
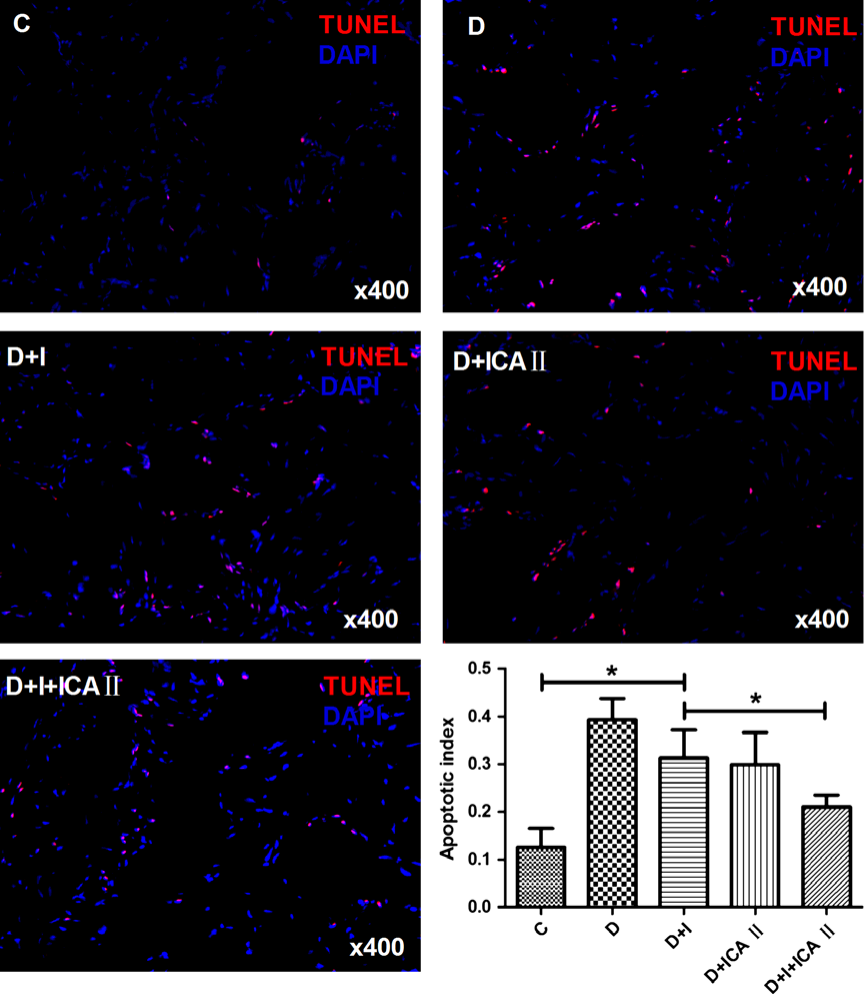
Evaluation of apoptosis with TUNEL staining. Representative images for TUNEL staining of corpus cavernosum tissue are shown. The nuclei of all cells were stained by DAPI (blue fluorescence). Apoptotic index is presented as the ratio of apoptotic nuclei to the total number of nuclei counted. Combination therapy significant reduced the apoptotic index to near control levels. * denotes *P* < 0.05 when comparing the two groups under each end of the capped line. C, control group; D, diabetic group; I, insulin group; ICA II, icariside II.

The apoptotic index (percentage of TUNEL-positive cells) was significantly higher in the diabetic group compared to the normal group. Insulin monotherapy was able to reduce the apoptotic index to some degree; however, there was still a significant difference in apoptotic index between the insulin-treated group and the normal group. Combination treatment reduced the amount of TUNEL positive cells to near normal levels (Fig.7).

## Discussion

Although DM can be controlled through medications, diet, and lifestyle modifications such that blood glucose levels may be normal, patients with DM continue to be afflicted with numerous complications, including ED. In addition to the significant morbidity and mortality associated with these complications, there is a marked reduction in quality of life. Large randomized studies have established that delayed intensive therapy fails to reduce mortality and major cardiovascular events in type 2 diabetic patients. In fact, patients who previously were not managed with intensive glycemic control demonstrated worse outcome compared to patients whose glycemia was controlled 16,17. Follow-up data from these trials reveal an influence of early metabolic control on long-term outcome in these patients 18. This phenomenon has recently been defined as ‘metabolic memory’. Binding of AGEs to RAGE results in generation of Reactive oxygen species (ROS), with subsequent activation of the redox-sensitive transcription factor nuclear factor-κB in cells of vessel walls, which promotes the expression of a variety of atherosclerosis-related genes and RAGE as well 19.

Cho *et al*. showed that tight glycemic control was able to restore ICP/MAP to the level observed in normal control rats. Moreover, they found no difference in apoptotic index between the tight glycemic-control group and the normal group 13. However, according to the results of our study, tight glycemic control did not completely restore erectile function and apoptotic index to normal levels, possibly because of the previous exposure to elevated blood glucose levels, demonstrating the metabolic memory phenomenon.

Wei *et al*. showed that AGEs can accelerate rat vascular calcification through RAGE/oxidative stress 20. Oxidative stress is characterized by an imbalance between the production of ROS and the endogenous antioxidant defences at the cellular level. Increased oxidative stress in cavernous tissues not only compromises nitric oxide (NO) bioavailability, which is necessary for erectile function 21, but also induces injury to the cavernous cells.

Icariside II is a type of flavonol isolated from herba epimedii. Although there is a small amount of ICA II in natural herba epimedii, ICA II can be transformed from icariin by intestinal bacteria after oral administration. ICA II can also be generated from icariin through the action of β-glucosidase *in vitro* 22. Lui demonstrated that icariin has the antioxidant capacity to protect linoleic acid against free-radical-induced peroxidation in micelles 23. SOD is a key enzyme in the antioxidant response. Additionally, MDA is an indicator of lipid peroxidation by ROS. We showed here that the combination of ICA II and insulin led to increased SOD expression and decreased MDA expression, nearing the levels observed in control non-diabetic rats. We also demonstrated that insulin plus ICA II resulted in improvement of erectile function greater that than produced by insulin or ICA II alone. Moreover, this combination therapy restored erectile function to the level recorded in non-diabetic control animals. We also demonstrated that combination therapy led to increased content of penile endothelial cells and smooth muscle, and nNOS-expressing nerve endings, all of which approached the levels observed in control non-diabetic rats. These histological changes were greater in the combination group compared to either the insulin or ICA II monotherapy group. Thus, the ongoing progression of diabetic complications despite normalized blood glucose values may be halted by anti-oxidants such as icariside II 10.

A shortcoming of this study is that STZ-induced diabetes is an artificial form of DM, closer to an animal type 1 DM model than to type 2 DM, the latter being a far more common cause of morbidity in humans. Although we identified clear histological and molecular changes that play a role in improvement of erectile function, there may be other factors that we are not aware of that may mediate this improvement in function as well.

## Conclusions

These results suggest that, although insulin monotherapy controls blood glucose levels effectively, it is not able to completely alter the pathological changes in erectile tissues and does not preserve erectile function. Better efficacy could be expected with tight glycemic control plus icariside II, which has antioxidant effects. The combination therapy might have the potential to eliminate metabolic memory by down-regulating the AGEs-RAGE-oxidative stress axis.
